# Emerging athletes’ career transitions in professional sport: an existential multi-case perspective

**DOI:** 10.3389/fspor.2024.1401848

**Published:** 2024-07-04

**Authors:** P. G. Thomas, P. Lucas, S. Walters, A. R. H. Oldham

**Affiliations:** Sports Performance Research Institute NZ, Auckland University of Technology, Auckland, New Zealand

**Keywords:** athlete, professional sport, boxing, rugby league, basketball, athlete careers

## Abstract

**Introduction:**

This article examines athletes preparing for, transitioning into, or going through the developmental stages of a professional sports career, referred to as the emerging athlete career transition. This transition includes events such as selections, Junior-to-Senior promotions, contracting, migration, and early exits. The article presents the collective findings of a multi-case study in three professional sports: rugby league, basketball, and boxing.

**Method:**

Consistent with pragmatic qualitative research methodology, a stratified data set was collected and analysed, incorporating researcher-practitioner fieldwork, interviews, documents and artefacts for these cases. This article explores unique events and the demographic and cultural implications of navigating emergent transitions along professional sporting pathways in New Zealand.

**Findings:**

These findings highlight the importance of building self-efficacy as a pre-condition for coping through preparation and experience.

**Recommendations:**

Recommendations include fostering collaborative cultures and authentic support relationships to facilitate better coping alongside athletic and personal development in these high-pressured environments. Furthermore, understanding existential perspectives of meaning, choice, and responsibility provides insights for developing the resources that allow emerging athletes to thrive in life beyond sport.

## 1 Introduction

Professional^1^ sport is a relatively new phenomenon which has increased in size and sophistication in recent decades, as seen in this lucrative industry around ticket sales, media, and endorsements. In the US alone, this market was recently estimated to be 64.8 billion USD (1). This article explores unique events alongside the demographic and cultural constraints of sports career transitions experienced along professional pathways in Aotearoa, New Zealand (NZ). While obtaining robust financial metrics in NZ is difficult, significant investment in various professional sports through public and private funding channels exist. Professional rugby union, for example, given its cultural significance and popularity in the country, attracts substantial financial investment. However, unlike the US, NZ does not have a robust collegiate pipeline for any professional sport and endorsement networks are limited. NZ's geographical size and remote location often necessitate the relocation of athletes to colleges, clubs or promotions outside the country. The region's Māori and Pasifika^2^ peoples are frequently sought after worldwide for their physicality and perceived warrior-like characteristics (3). This widespread relocation of athletic communities, which involves collectivist cultures, offers different discourses on how individuals see performance, family and community. Dichotomies of roles and identities present challenges to authenticity when confronting professional sports codified and contractual demands. These issues offer many questions of interest from an existential perspective concerning choice, responsibility, meaning and relationships.

**Figure 1:**
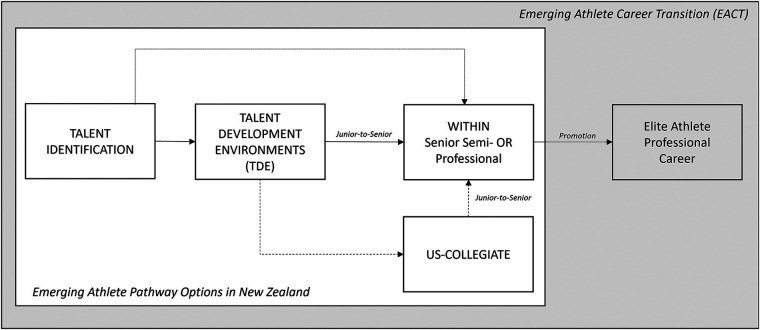
NZ pathway environments and phases in the emerging athlete career transition.

This article's scope sits within the athlete career discourse by examining athletes preparing for, transitioning into, or going through the developmental stages of a professional sports career, as depicted in Figure 1. These athletes are referred to as *emerging*. The *emerging athlete career transition* (EACT) commences with talent identification by a *scout* (club, agent or promoter) who may recruit the identified athlete as a *prospect*. The prospect then moves through multiple iterations of selection “*phases*” (4) within different organisational or collegiate talent development *environments* (TDE). TDEs are designed to build athletic performance (5). The EACT concludes (if successful) with promotion into professional apprenticeships via the Junior-to-Senior phase. The reality, however, is many athletic careers end during this process, having barely started. Athletes who fail to progress to the next phase through non-selection (not being selected into TDE after identification), de-selection (selected and then rejected), withdrawal (self-removal), injury (season/career ending) or eligibility expiry (failure to meet collegiate criteria or graduation) are termed “*early exits*”, defined as the “premature termination of a sports career prior to reaching their peak performance” [(6), p. 485].

This article consolidates findings of a multi-case study doctoral project in rugby league, basketball, and boxing, using an existential lens to examine how relationships facilitate or hinder this transition and the events that occur within it. Identifying and understanding participants' roles in their professional sports environments assists with interpreting patterns within sporting relationships. Hence, this article addresses the following questions: (a) What events and challenges do emerging athletes experience during transition in professional sport? (b) How do existential relationships facilitate or hinder this transition?

### 1.1 Fundamentals of athlete career transitions

Research in athletic professions positions career transitions as an ongoing coping process (6–8). This article explores EACTs as a process of coping with dynamic and overlapping events with varying degrees of expectancy, intensity and controllability (9). Examples of events include selection, migration, and coach conflict. Depending on many factors, athletes who experience setbacks, such as selection failures or minor injuries, may still rebuild and return. When an individual is threatened, the autonomic nervous system initially triggers fight, flight, freeze or fawn (appease) responses (10). While not all events are adverse, all events require the individual to react. The individual responds by assessing the stressors for personal context, determining resource availability (*appraisal*), and then adapting, contributing to facilitative or crisis *outcomes* (11). *Adaptions* will depend on the athlete's appraisal of *time* (age, career stage, prior exposure), *context* (event accumulation, experience, predictability, controllability, repercussions, and personal priority) and available *resources* (tangible, internal and external support) (11–14). Coping strategies include *problem-focussed* (cognitive, action), *emotional-focussed* (outbursts or avoidance) or help-seeking (11).

Over recent years, researchers have sought to expand the athletic career discourse, extending scholarly focus on sporting career transitions and events (15). Published articles in EACT were predominately limited to football (soccer) and rugby league, with relatively little identifiable literature on boxing (16), basketball (17), and collegiate EACT (18–21). Researchers conducted seven EACT-relatable systematic reviews since 2014 (9, 15, 22–26). The articles reviewed were commonly athlete-centred, whether written from the athlete's or one stakeholder's perspective, across a short time window, focussed on Junior-to-Senior, or used instruments (27) to evaluate TDE sporting environments. Single-actor analyses may skew findings. For example, when an athlete is dropped from a programme, they may fail to take responsibility, blaming the coach or the process. Furthermore, given EACT span years, using snapshot time data will reduce efficacy. As such, research expanding sports environments over longitudinal timeframes provides a more informed perspective on career transitions. Notable EACT literature included multiple sports (26) or other stakeholder perspectives in rugby league (28–30) and transferrable articles in soccer (31–36) and hockey (37, 38). Readers can find an in-depth literature examination in the contributing thesis (39).

**Figure 2:**
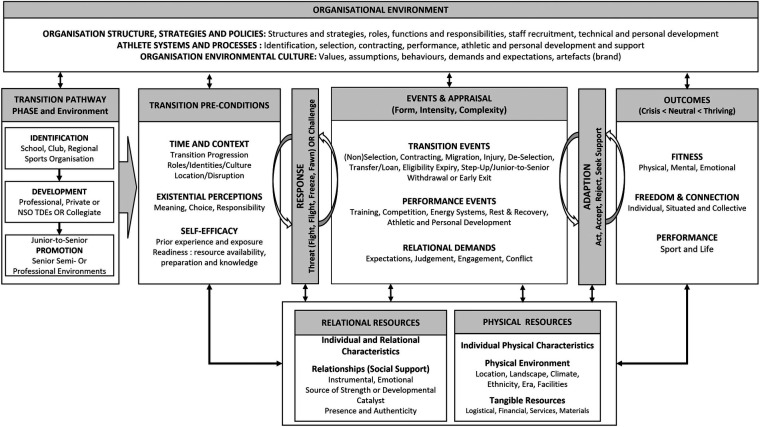
Proposed conceptual model for coping in emerging athlete career transitions (EACT).

This work expands existing epistemology, offering a multi-stakeholder, longitudinal approach to understanding the dynamic balance of relationships and systems that support successful transitions. It also recognises an athlete's responsibility for support-seeking and building resilience (40). Stambulova et al.'s international review called for “special efforts” to be made by the research community to transform and expand the currently developing knowledge in the athletic career discourse, including diversity of environments and cultures, development, transition, and assistance (15). Additionally, they stated the need for models to be in a “form that allows athletes, coaches, parents and other stakeholders to understand and benefit from” (p. 18). This article responds to this call. The proposed conceptual model presented in Figure 2 extends the models in previous articles (22, 41, 42).

### 1.2 Existential philosophy and psychology

The article examines EACT using an existential framework. Sartrean existentialism identifies identity, meaning, freedom (of choice and consequential responsibility), isolation and death as the “Big-5” existential concerns (43). Positioning EACT alongside existential philosophy examines athletes' and other stakeholders' experiences of choice and responsibility, identity, meaning, and relationship, with death translated to transitional events and endings. Identity was further interpreted as self, social (25, 44, 45), identity fusion (46) and athlete identity foreclosure (47, 48). Significant existential anguish in athlete transition emerges from career setbacks and endings, where being in the world only has meaning in so far as it opens towards a possible future (43). Forgoing one opportunity over another is particularly pertinent to athletes, where dedication to a sporting ambition involves “setting aside and devoting oneself, one's time, one's body” to their sports project [(49), p. 37]. Professional sports must represent a meaningful project to enable the individual to endure the pressures, limitations and risks inherent in such a career choice (49).

Furthermore, we cannot divorce ourselves from the world (43). Individuals exist in s*ituated freedom,* as physical and environmental facticity's and institutional regulations bind them; organisations^3^ regulate the structure, processes, and cultures they engage in (43, 49, 50). Despite this, individuals desire authenticity and connection in their relationships (50). To be authentic is to act in good faith, true to our own identity and meaning, as we seek to avoid isolation and loneliness (43). To be connected is to see and respect the humanity in another (50). Therefore, existential philosophy, as applied in existential psychology (51–53), provides a robust framework for examining events and relationships (self and others) for EACT.

### 1.3 Roles and relationships

While the central actor of this study rests with emerging professional athletes, other organisational figures (e.g., coaches, managers, agents), practitioners (e.g., trainers, physiotherapists, nutritionists) and external stakeholders (e.g., family, fans, etc.) contribute to paradoxes of demands, expectations, service and support in their sporting world. To understand relationships, we must examine both sides of the dyad beyond the role and mask (54). Roles are patterns of behaviour or actions expected of an individual within a social setting (55), a role arguably representing one aspect of the individual's social identity (45), alongside individual facititys (e.g., age, gender). With athlete wellbeing an increasing concern for sporting bodies, research has moved into examining broader practitioner roles (e.g., sports psychology, player welfare) and defining the construct in talent development environments (5). During the emerging transition, adolescents are not only faced with a series of events and demands (56) but are typically undergoing physical and psycho-social development (8, 57). This development involves constant review and modification to existential assumptions, deciphering who and why they are (identity, meaning, authenticity), the roles they fill (e.g., athlete, son, teenager) and the choices and responsibilities they take (e.g., train or party) (49). Hauser et al. argue emerging athlete environments should offer greater opportunities to nurture adolescent athletes' long-term personal growth and athletic development (28).

Emerging athletes use social support relationships to cope with demands and events throughout their career transition (58). These relationships fall into instrumental or emotional categories (59). *Instrumental* support is received via tangible supply (e.g., provision of additional equipment) or information (e.g., knowledgeable advice on contracting). *Emotional* support involves a listening ear, a place to vent or a boost in the athletes' esteem. To investigate these relationships, we adopted Feeney and Collins (59) framework, which extends earlier social support theories (60, 61) by linking the construct to well-being and thriving. When thriving, individuals have positive psychological (cognitive, emotional), sociological and physical well-being, including meaning, sense of achievement and connection with others. Feeney and Collins propose social support relationships are not simply a resource for coping with adversity *but* a facilitator for growth and development. Equally, they argue the support receiver is *responsible* for shaping their support outcomes, for example, by reaching out, being receptive to efforts, not over-taxing demands, and returning gratitude and support. Furthermore, the supporter and recipient are entitled to mutual benefits. We anticipated that understanding the influence (facilitative and debilitative) of relationships, including the athlete's relationship with themselves, would offer greater knowledge and understanding of how to facilitate EACTs.

## 2 Methods

The lead author undertook the research as an independent researcher-practitioner in *active service* with emerging male athletes transitioning into professional sports in the three case sports. The research objective was to design the research to examine issues and concerns experienced by the researcher-practitioner for the athlete cohorts she was involved with (62). This design method offers a greater chance of observing an individual's expression of meaning, non-verbal references, deliberate and/or inadvertent actions and behaviours and/or protective responses in real-life, real-time in their natural environments (63). The role of the professional athlete is often revered as that of a super-hero, selection predicated on performance and elitism. So, maintaining a public “face” in sports can be substantial. Being a trusted insider allowed the researcher to break through these personas to uncover the existential challenges the participants were experiencing. This relationship and shared experiences enabled a comfortable rapport for open conversations and trust to delve deeper into discussions during the interview stage. Managing the risk of over-familiarisation required rigorous awareness of when she was a practitioner, a researcher, or a friend, ensuring the authenticity of the practitioner role remained paramount. This included reflective interviews with an independent sports psychologist, her mentor and critical friends (supervisors). All participants were fully informed and understood the researcher-practitioner's involvement in her doctoral research, and due process was undertaken at all times. Full ethical approval was granted through AUTEC (19/395).

### 2.1 Research context and positioning

This study intended to go beyond capturing participants' views via surveys and interviews to examine the existential tensions between emerging athletes and their embedded network of relationships. The methodology uses a pragmatic paradigm (64) with epistemological views of reality defined by Maarouf (65). The ontological position adopted an existential view of being in the world (43, 50), and the research used a qualitative multi-case study methodology drawing on the work of Merriam (66) and Stake (67). The work of Aggerholm (49) provided existential insight into talent identification and development, with Nesti and Ronkainnen providing an existential psychological perspective (51, 53).

Collective instrumental case studies seek to accomplish insight into an issue and are designed to help refine a theory or facilitate the understanding of the subject area (62). Using a pragmatic approach alongside Maarouf's reality cycle, findings were positioned where multiple individual perspectives and behaviours can be presented as a “single reality” inside a defined time and context. This paradigm offers an opportunity to explain “common threads in the messiness of life” [(68), p. 7] by providing a systematic methodology to explore the similarities and differences within and between cases in anticipation of finding these patterns. Increasing the number of cases makes more information available to expand our understanding of the complexities of professional sports and support findings and recommendations for identifiable social problems (66, 67, 69). As pragmatic researcher-practitioners, we are responsible for facilitating beneficial solutions to the practical problems we encounter. Sourcing solutions required questioning participants about the environment and relationships (including one with self) they inhabit, the meaning they give, and the responsibility they take for their choices, actions and outcomes.

### 2.2 Research design and implementation

**Figure 3:**
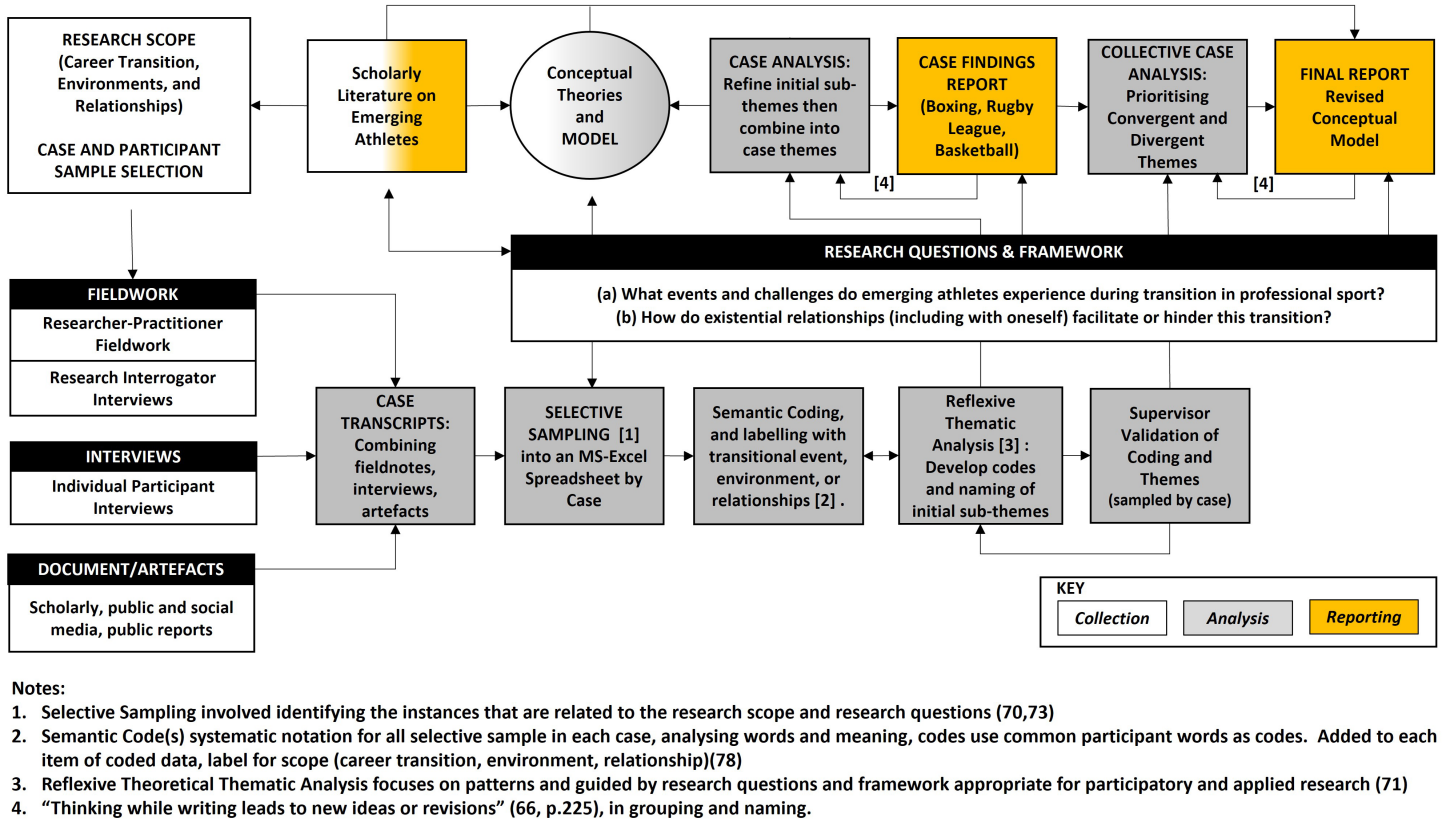
Research design and implementation schematic.

Figure 3 represents the research design method implemented, showing the steps to complete the collection (white), analysis (grey) and reporting (yellow). Within case study research, the researcher chooses what to study, each case can have a distinct context, and the data collected between cases can take varying forms (62, 67, 70). This approach is compatible with a pragmatic qualitative research paradigm, intended to identify patterns and solutions across cases and guided by the theoretical framework detailed in sections 1.1-1-3. The design implemented uses a systematic reflexive theoretical approach in analysis and reporting (71), recognising the importance of the researcher-practitioner's “subjectivity as an analytic resource and their reflexive engagement with theory, data, and interpretation” [(71), p. 3].

A longitudinal study was considered the best approach to investigate the complex nature of this subject matter, given transitions span years, with events occurring over weeks or months. Case-study methodologies require cases to have defined boundaries (62). This research is bound by professional sports environments and roles (context) across three sports: boxing, basketball, and rugby league (cases) over a 3-year transition period (time). The commencement and end of the data collection were simultaneous across all three cases, including 2–3 years active service fieldwork, with additional external observation of relevant participant events up to the reporting phase (detailed by case below).

#### 2.2.1 Case and participant selection

The sample of cases and participants was purposive, with convenience sampling conducted through the researcher-practitioner's contacts and connections within the sporting industry (70, 72). Stake emphasizes that cases should be chosen for their potential to provide rich information that addresses the research questions (62), with Gentles acknowledging that in case research the data collected within and between cases can take varying forms (70). Braun and Clarke stiplulate determining sample size in qualitative projects is a pragmatic exercise (73).

**Figure 4:**
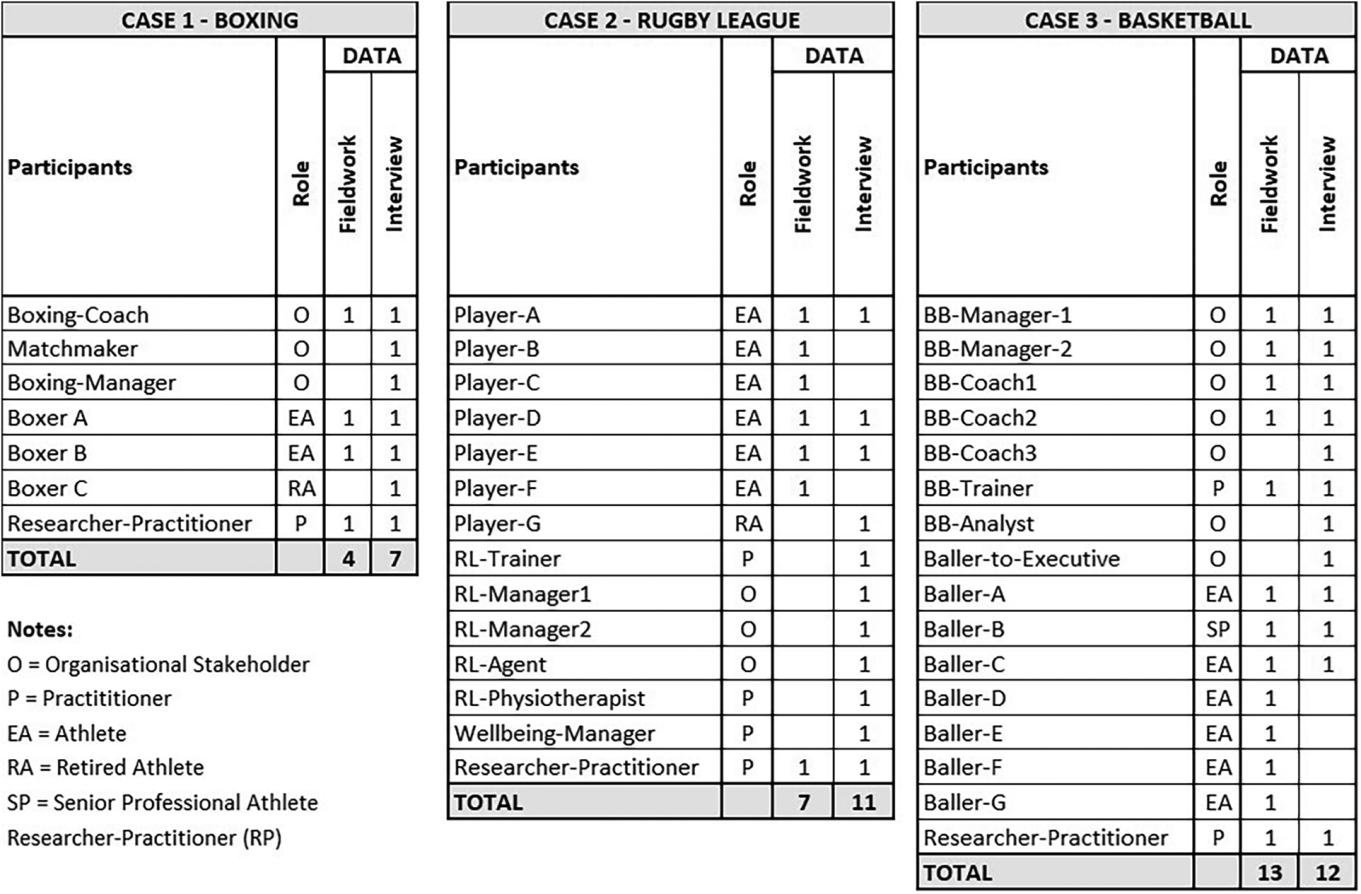
Summary of participants by case.

Participants were selected as best placed for their knowledge and insight into the phenomenon (purposive) and their convenience (accessibility, availability) and willingness to participate (72). The extent of case and participant selection in this study was limited to the researcher-practitioner's *active service* network and to the “time [and] financial constraints [on this] project” [(71), p. 11, 73]. Participants were included to embrace a broad range of events, roles, relationships and knowledge relevant to each case. Using the researcher-practitioner's network and involvement in the three case sports, allowed rare access and opportunities to observe this phenomenon in their actual (63). Figure 4 summarises participants across fieldwork and interviews involvement. The participant label and role indicate the position they filled. Additional demographic information is excluded to protect the participants' anonymity in a small geographical sample.

The following sections provide a brief description of the three sports cases.

##### 2.2.1.1 Case 1: Boxing

Boxing in NZ was established as a professional sport in 1902 (74). Open boxing is a three-round competition with points scored for connecting strikes. Professional boxing is typically contested over 8–12 rounds of fierce combat aimed at knocking the opponent to the canvas and out, with points scored for power punches. Unregistered *corporate* and/or celebrity boxers (initially with a training or charity focus) has now evolved as an alternative pathway for professional fighters. Furthermore, the demarcation of open boxing as amateur has now blurred with changes in international organisational body rules (75). In professional boxing, genuine “prospects” are contracted to promoters. Promoters organise boxing events that combine these contracted athletes (stable boxers) and outside opponents to match fights on cards, with each boxer receiving a purse (payment) to compete. Journeymen (seasoned boxers) are paid to compete against prospects or contenders with no expectation of winning. This case examines the researcher-practitioner's involvement with an emerging professional boxing team (Boxer-B) in years 1 and 2, with external observation in year 3, and an individual support relationship (Boxer-A) over the 3-year period. Retired Boxer-C was interviewed to broaden case participant experiences.

##### 2.2.1.2 Case 2: Rugby league

Rugby league split from rugby union in Britain in 1895 when players from predominately lower socio-economic backgrounds demanded payment to play. Rugby league then diverged from rugby union into a higher-paced, heavier-contact sport to enhance spectator entertainment and contribute to players' incomes (76). This case is in the Australasian National Rugby League (NRL), comprising 17 clubs, including the NZ-based Warriors. NRL clubs now contract players to TDEs from 15 to 16 years. This case examines the NSW pathway, commencing from Harold Matts (junior competition 16–17-years) to Junior-to-Senior transition (typically 20–23-years). Case 2 involved 3-years active independent practitioner service (through her academy or individual support relationships) with Players A-F. Players A-F were involved with several different NRL club environments during this time. Player-G, a previous client was included in the interview phase.

##### 2.2.1.3 Case 3: Basketball

Basketball in NZ provides professional and semi-professional opportunities for players in the NZ National Basketball League (NBL) and the Australian NBL, with pathways via overseas leagues such as the US-based National Basketball Association (NBA) and US-Collegiate systems. This case involved independent service with Baller-A and BB-Coach-3 for 3 years. The case also included 6-months wellbeing support with an NZ-NBL team mid-year-1, with the researcher-practitioner continuing support and/or correspondence with participants for the remaining study period [(71), p. 11, 73].

#### 2.2.3 Data collection

The stratified data collection method combined three sources: fieldwork, participant interviews and document/artefact review, enabling the authentication of ideas and interpretation and triangulation of fieldwork data (67, 73). Fieldwork collection involved recording the researcher-practitioner's experiences during active service with athletes and interactions with other stakeholders in the three sports codes over 3 years. Documenting observations, combined with action (working, joking, hanging out), correspondence and personal lines of questioning (of self and others), provided objective and subjective data. The researcher-practitioner recorded detailed observations and conversations during periods of active service.

Participant interviews followed active service engagements were purposive to exclude athletes still in active service and to ensure individuals' vulnerabilities were protected, and ongoing practitioner support was not compromised (72). Interview invitations included a broad coverage of the various roles (athlete, coach, manager, trainer and practitioners) and a wide range of events and career progressions. All invitations to interview were accepted. Audio-recorded and transcribed interviews were face-to-face or on Zoom, necessitated by the ever-changing environment during a pandemic and the convenience of participants. The approach was conversational, and the participant was encouraged, first and foremost, to tell their stories. The researcher-practitioner designed the interviews to address the two research questions. The interview framework was provided to participants in advance: (1) roles, identity, and authenticity; (2) relationship with others; (3) freedom and responsibility; and (4) coping with transitions and events. The interview approach adopted the engagement principles of existential psychology (51) and Roger's principles of unconditional positive regard (77).

In addition to scholarly literature, the researcher-practitioner collected publicly available media (athlete non-fiction, public reports, online news media, internet Google searches, YouTube videos, podcasts, Facebook, and Instagram posts) applicable and/or publicly available for any of the participants or cases.

#### 2.2.4 Data analysis

**Figure 5:**
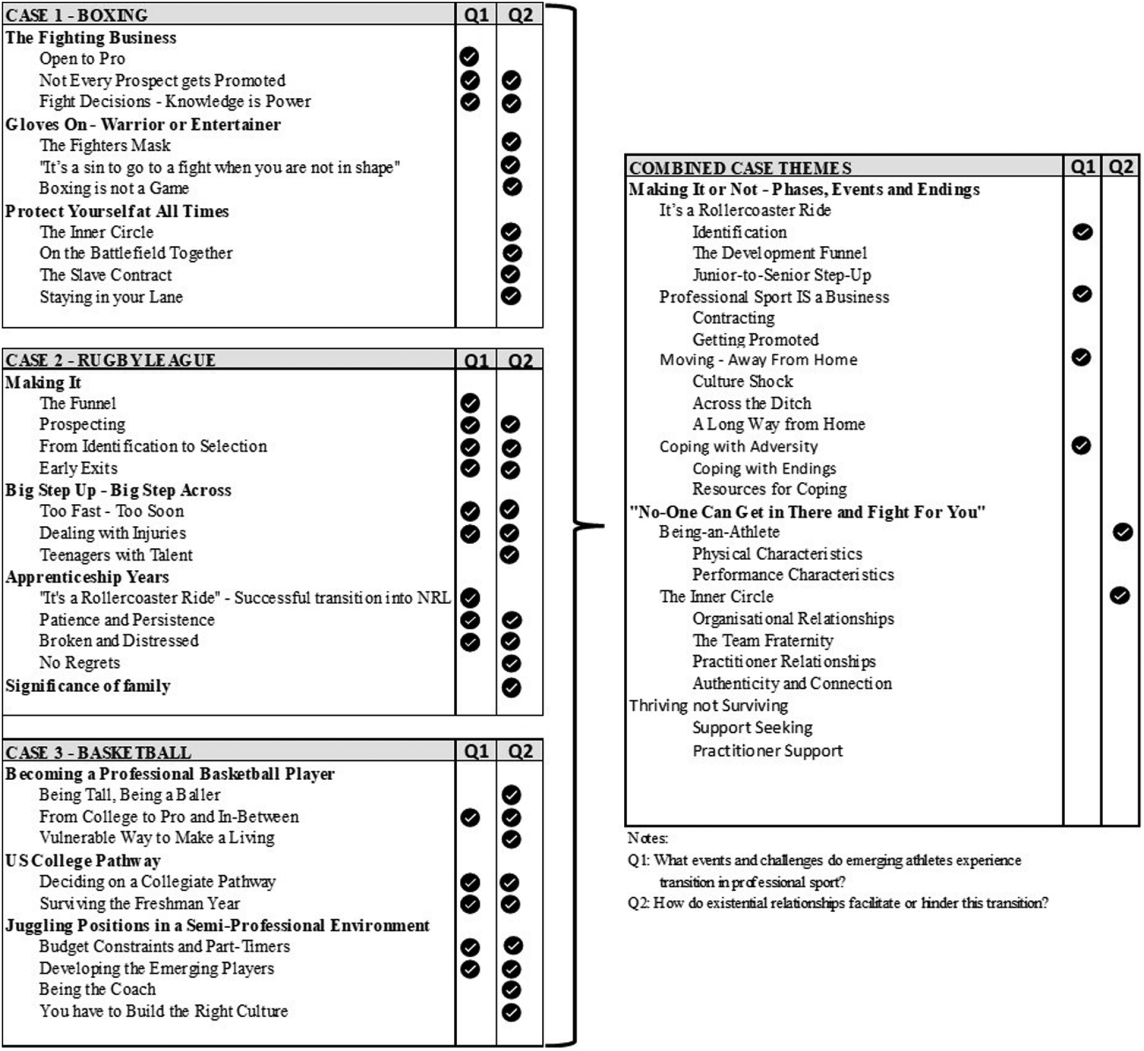
Summary of case and combined themes.

Figure 3 details the reflexive theoretical thematic analysis approach (71, 78) used in this research. Analysis commencing by combining all three data sources into a case transcript. The researcher-practitioner then re-familiarised herself with the stratified case results, adding reflection notes where appropriate. Prominent samples were then extracted into a spreadsheet. Each case was semantically coded by the researcher-practitioner using keywords, sentiments or phrases identified. The researcher-practitioner then reflexively applied sub-themes by merging similar codes and ideas prioritised by the research questions, combining the sub-themes into themes (Figure 5) in each case report (66). This process was repeated for all three cases, with independent sample-based validation carried out by a supervisor. Case themes were compared and evaluated for convergent and divergent themes, with a decision to modify, add, or delete themes into the combined themes and final report. The initial conceptual model, informed by previous research (14, 22, 41, 42), was modified, adapted, and refined throughout the study.

## 3 Collective case findings

The following sections discuss the collective findings under two themes (Figure 5): “*Making it or Not*”, responding to question 1, and “*No-one Can Get in There and Fight for You*”*,* question 2.

### 3.1 “Making it or not”

Despite the length, complexity and intensity of EACTs (especially when identified early), RP noted athletes were always impatient to step up faster, thinking they could “make it” quicker (79). With this impatience and/or youthful perception of time, athletes generally knew what was required but “often did not know the process” (RL-Manager2). That is, they had sufficient awareness of the athletic responsibilities (commitment and hard work) (53) but did *not* understand the intensity, the length of time it would take and the cost. More critically, individuals often lack the necessary knowledge of the sports business and the brutal probabilities of their success. From selection to career end, coaches, selectors, and promoters closely monitor their every move, exposing athletes to constant criticism: “imagine your boss sitting over your shoulder every day, watching your every move” (Baller-to-Executive). The scenario reminds us of Sartre's famous quote, “hell is other people” (80), as we are forced to see ourselves through the eyes of others.

Furthermore, identification processes often involved “blowing smoke up their butt” (Wellbeing-manager) or athletes and their families being “sold false dreams” (RL-Manager1) (28). When young men are recruited or selected for coveted sporting positions and endowed with dollars and apparel, it is unsurprising their egos get inflated. Complacency and entitlement appeared to be a greater risk for rugby league players on higher-value entry contracts than players striving to gain positions (81). Athletes who experienced adversity earlier (e.g., not picked or faced with an injury) developed better coping capabilities and hunger to succeed (82). “Initial stars” having “[signing] that piece of paper, [it was] tools down, I’m sorted” (RL_Manager2) and “Players who [hadn't] been told the truth in the past … think they are better than they are” (BB-Manager2). Sadly, for many, their sporting careers ended before they even started, an existential truth the young people in the case studies were unaware of or needed greater maturity to accept.

The following sub-themes examine participant experiences of EACT phases and events involved in “*Making it or Not*”.

#### 3.1.1 It's a rollercoaster ride

In Cases 2 and 3, the EACT typically coincides with navigating adolescence, “full of hormones and short-tempered” [(83), p. 181] and involves a series of phases from identification, development in pathways environments and an “apprenticeship” period called Junior-to-Senior. In boxing, the transition differs. All three boxers (case 1), Boxer-B and Boxer-C having entered the sport considerably late at 19 years, were still in the EACT into their late 20s. The professional boxer will remain a prospect until they reach top-15 in world rankings (Matchmaker).

##### 3.1.1.1 Identification

From identification, the athletes commence an ongoing cycle of selections for progressions and playing positions (4). Identification events and processes differed between the two team-sport cases (2 and 3) and the individual sport of boxing (case 1). All three sports rely on scouts to identify athletes with potential and talent. Typically, physical requirements (sports skill and athleticism) are the primary credentials for identification and progression for a prospective athlete. However, early identification at pre-adolescence can misrepresent an individual's potential, particularly when solely limited to these criteria (79, 83). Being the tallest, biggest or strongest at a young age is not a blueprint for future success because others mature and develop later (RL-Manager1^4^). Furthermore, these physical characteristics do not always imply desire, commitment and psycho-social acumen. From identification, someone *then* needs to teach them to behave and train to be an athlete (RL-Trainer, BB-Trainer), transforming them from having-a-talent to being one (49).

For the boxer, identification and progression are less definitive, commencing with the boxer/team's decision to transition. However, Matchmaker emphasised, “I have seen excellent boxers with stellar amateur careers who couldn't cut the mustard as pro-boxers”.

##### 3.1.1.2 The development funnel

From entry into pathways environments, the athletes continue an ongoing cycle of selections for progressions and playing positions (4). In the two team sport cases, athletes enter the TDE funnel, which filters future contracted players from identified talent over time. In rugby league, promotion through the junior pathway is a series of steps; as RL-Manager1 explained, “SG-Ball is a big step. It's another big step up to Flegg and an even bigger step to NRL”. The emerging basketballers playing on the semi-professional NZ-NBL side were unpaid, arguably to retain the required “amateur” eligibility for collegiate placements. Baller-D and Baller-E excelled in the side, playing significant minutes; however, for Baller-C, it was frustrating: “I came into the team expecting to play a role” but only getting a few final minutes in already won or lost games “just ruins your confidence”. Adolescent athletes moved from environments where they enjoyed playing with friends they had grown up (Player-C) with to team-mates who became rivals (84). Players spoke of “ruthless” cultures, comparing themselves to others and trying to convince themselves they were good enough because “no one is weaker than you; everyone is good” (Player-A), and feelings of isolation, “the person going to look out for you most is yourself” (Player-D).

Emerging athletes regularly struggled with playing status subjugation, like the rugby league players going from a “big-fish in a little pond” (RL-Manager1), the frustrated ballers not “getting minutes” on the court; or the Division-I freshman “I'll go to someone who really wants me”. His father (BB-Manager2) explained, “He needs to learn how to handle things; I can't interfere.” Despite apparent talent and intense training, athletes still did not make it into competition teams or “sat on the bench” (e.g., Player-B, Baller-F). Sidelined athletes capable of coping with these subjugations earned the coaches' respect, enhancing future selection chances by “showing they're committed to the group” (BB-Coach1). Those who failed to maintain these qualities either quit (Baller-F), remained on the outer (Baller-C, Player-E), or were deselected from the programme (Baller-G). These findings concurred with previous studies (30, 31, 37, 38).

The year after leaving high school was particularly challenging for athletes in this transition phase, adjusting to impending adulthood and their sports apprenticeship (RP). The NRL regulates a “no study, no work, no play” policy (85), which often involves a physically demanding job (e.g., labouring) and/or tertiary education alongside training commitments. RP evidenced players battling with training commitments and energy levels and lacking real effort towards vocation and/or education commitments. Arguably, the collegiate pathway provides athletes with a system to study and play in one institution. However, this pathway is closed for non-academic athletes who fail to meet eligibility requirements. Furthermore, student-athletes are still exposed to extensive hours of training and playing alongside academic requirements (20, 86). Regardless of the pressures, RP witnessed the athletes having entered TDEs as adolescents demonstrably maturing physically and psycho-socially (8), becoming more independent, accountable and stretching their boundaries beyond parental inputs (32, 87, 88). This growing independence was also seen as an uncomfortable transition for parents, notwithstanding a necessary one for the athlete, especially when it involved leaving home and going overseas.

##### 3.1.1.3 Junior-to-senior step-up

The Junior-to-Senior phase is when the *apprenticeship* period intensifies, the stakes rise, and tangible contracts are made or lost. In basketball and rugby league, by 20–23 years, athletes are either in or completing the Junior-to-Senior step, with a successful transition, *or not*, into senior professional careers before the age of 25. The percentage of prospected athletes who go on to “make it” into senior professional contracts is minuscule (R-L-Manager1, BB-Baller-to-Executive, Matchmaker).

Once in the professional arena, boxers must prove themselves through a series of fights. The lead-up fights are orchestrated for genuine prospects to win. Not every boxing prospect gets promoted, and until they do—there is little money to be made (Boxing-Coach). Promotion requires proof of the boxer's athletic abilities and entertainment value. Losing during this period destroys contender^5^ opportunities (e.g., Boxer-A). In rugby league, promotion to senior environments “is a really big jump, intensity wise and hours wise.” (RL-Manager1), with boys training and competing alongside full-grown men “who don't go soft on you” (Player-D). Contracts depend on individual performance and impressing coaches and organisational representatives. Athletes across all sports expressed feeling the pressure to impress those around them. For example, Player-A, who accelerated to NRL at 19, said, “I felt pressure … you want to impress … just being a newcomer.” Player-D spoke of senior players making him feel part of the team, “but when they speak, you listen”. Player-A referred to feeling awkward with the head coach, while Player-D referred to being intimidated, “he scares the s#*@ out of me … he's got a good blowup in him”.

Significantly, Player-A's early Junior-to-Senior selection and injury events were a rollercoaster ride tested his patience and resolve, but he persevered (40). Similarly, Baller-A returned to NZ after graduating from his US-college and entered the NZ-NBL semi-professional environment. He said, “I felt like the coaches were looking at me … people were curious to see what I had become … I didn't really know how to behave”. Baller-to-Executive observed, “He came back from the US with something to prove; this tightened up his game, and he ended up on the bench”.

#### 3.1.2 Professional sport is a business

Participants stepping into professional sporting pathways quickly appreciate they are in a business: “It's simple, it's business. It is your livelihood” (Boxing-Manager), “You've got to have something to be in a team … from there, it's business” (Player-G), “the business is real—is all gambling” (Boxer-A). “When people are paid to play … there are different dynamics involved … they want their money in the bank” (BB-Analyst); however, individual interpretations of being in the business differed. Player-A and Player-G, understood their role as business resources but denied feeling like a commodity, reflecting a love for the sport and “representing the club”. However, basketball participants described it as “a vulnerable way to make a living”, “100% a commodity” (Baller-B) and getting “paid according to what the coach thinks you are worth” (Baller-to-Executive). The variation between the two sports is inconclusive; differences likely reflect participant time and context, bound by individual exposures and experiences, and the greater transience of seasonal professional basketball contracts.

##### 3.1.2.1 Contracting

Participants referred to scouts “whitebaiting”^6^ casting a wide net in the hope of signing that one exceptional athlete, “it's a numbers game … forget about the ethical side, from a commercial point of view, you can't do a proper job for them” (RL-Agent). Recruitment into US-Collegiates varies significantly depending on the divisional level of the institution. BB-Analyst explained that “unless you're in the top 2% of players in the country”, opportunities will be scarce, but “if you're good enough [college scouts] come asking” (BB-Manager2).

RP found rugby league athletes and their parents were very aware of scouts prospecting at tournaments. However, unlike the mother in Storm's paper (84), distressed at having a monetary value set on her son, rugby league parents panicked their sons weren't good enough or their 14-year-old son had missed his chance. When parents were presented with contracts, they feared they would miss out or be penalised, if they didn't sign. After signing, parents proudly post club or agent agreements across social media, advertising they had “made it”. However, in truth, signing “agreements” merely “took them off the market” (RL-Physiotherapist, RL-Manager1) protecting the benefactors' interests and priority over their counterparts. The family typically received a nominal financial amount, with minimal support, in return for accepting this opportunity cost (RP). Furthermore, athletes and their families were observed negotiating and signing contracts they were ill-informed or unprepared (89). Athletes considered more talented by recruiters received full collegiate scholarships in top colleges and more significant financial remuneration in rugby league contracts (RP). Packages in rugby league included playing fees, education, medical and living-away-from-home allowances, with contract values becoming more lucrative as players enter semi-professional and professional environments. The NRL (Case 2), Australian NBL and NZ-NBL (Case 3) have stipulated regulations and salary caps for senior playing contracts, with agency agreements controlled by the Rugby League Players Association (RLPA) in rugby league. Surprisingly, junior contracts were not well governed during the data collection period. The NRL were the only organisation with an established athlete association at the time of writing, but the RLPA appeared to be predominantly focused on senior contracts.

##### 3.1.2.2 Getting promoted

The boxing business was considered difficult to get to grips with: “no one was telling us how to do it” (Boxing-Coach), and you cannot “buy a bag of experience on the way down to the boxing ring” (Matchmaker). Boxer-A who said, “People I trust so much who was leading me … I thought they knew what they were doing,”. Getting a professional boxer's career off the ground requires a slow build-up of fights and self-funding by “using my credit card and f@##### spending money on it” (Boxing-Manager). Without an influential and trustworthy promoter, the boxer will not reach championship bouts, and “if a promoter brings you in … he's not there to help you out” (Boxing-Manager). In boxing, purse values are at the promoter's discretion, with negotiations between the Matchmaker and the boxers' team for positions on fight cards. “I only got US$x … only paid to feed” (Boxer-A). Having the wrong person and/or signing the wrong contract “kept [Boxer-A] handicapped”. In an unguarded moment, Boxer-A stated, “it was a slave contract” (90). It is important to note here that Boxer-A took full responsibility for signing his management contract: “It's a dirty business—but my will is intact.”. Rightfully, the boxing community is calling for better organisational protection for their athletes (91).

All three cases demonstrated instances where emerging athletes were at risk due to a lack of knowledge and understanding of the business. Firstly, when there was a misrepresentation of their true talent and genuine chances of success, and secondly, how, when and what to contract for. Athletes (and parents) sometimes failed to understand the dyadic nature of relationships and the expectations of and commitments to fulfilling contracts. Conversely, the deferral of personal responsibility and/or an unreasonable burden placed on the coach and agents to provide services and levels of support can sometimes be unrealistic and unappreciated (31, 35). When contracts are not forthcoming, often the athlete has “failed to do their part” (RL-Physiotherapist) and “sometimes the players” expectations of the manager are wrong. They think they need to do everything for them” (RL-Manager1). The same applies to other roles: “Is it worth it? … we're the ones who choose [so] the ones to blame” (Boxing-Manager).

#### 3.1.3 Moving—away from home

Pursuing professional sporting careers from NZ regularly demands international travel or a move overseas. For professional boxers, training and fighting outside NZ is necessary for success because: “There is no money in boxing in NZ” (Boxing-Coach). Furthermore, unpromoted fighters entering overseas promotions as under-dogs means the promoter “does not want to see you win” (Boxing-Manager). With only one NRL club in NZ, rugby league players must travel to Australia for competition or migrate for contracts, and elite NZ basketballers typically move to US-Collegiates and/or travel to play in Australia or overseas leagues (BB-Manager1). While the necessities for moving differed, meaning and resiliency determined athletes' abilities to cope and/or thrive in their new environments (RP). Being away from home demanded adolescent athletes “grow up quickly”, behave maturely and make adult decisions young (30, 84). Moving away from home to pursue their dream, was referred to by some as the “sacrifice required” (92).

##### 3.1.3.1 Culture shock

Participants spoke of adapting to the environment, culture, intense coaching styles, expectations, and playing methods. The coaching and culture were more abrupt and competitive than athletes were used to, noticeably for Māori and Pasifika youth. “The Kiwi^7^ culture, particularly if you are Māori or PI, is to be more humble, but in the US, they see this as a weakness” (Baller-A), and “Australian juniors, they're way more competitive … a lot of the Polynesian boys … don't want to be yelling at their mates and stuff” (Player-D). Homesickness was a feature for all overseas participants at times, feeling isolated from home, friends, and family. Differences in seasons mean NZ US-College student-basketballers found themselves alone at Christmas (when Kiwis take their summer holiday), “sitting in the cold looking at screens of their mates having parties at the beach” (Baller-to-Executive).

##### 3.1.3.2 Across the ditch

RP evidenced adolescents, in Case 2, as young as 16, with or without contracts, sometimes with the family, sometimes alone, moving to Australia to vie for “better” perceived opportunities.^8^ Depending on the family, organisation, and contract, players stayed in a club residence, flatting situation, or were hosted by a family (38). Moving across the ditch was too soon for Player-E, who felt forced to leave before he was ready (84, 93). Regardless of blood connections, positioning players with family was not always constructive: “Going into a new family and not knowing them, it's really awkward … the chemistry is not there” (Player-E). Despite initial struggles to cook, clean, make friends and find their own way, many, like Players A and B, adapted and thrived (34, 42). Even for those well-prepared, the move was challenging (e.g., Player-D). Facilitative athletes found these challenges rewarding. For example, Player-A enjoyed “learning to be independent” and having a sense of “freedom”. Player-B, refusing to give up on his goal, strived to achieve success for his family. His mother told RP, “he always says everything is good, Mum; I never hear him saying anything he's homesick”.

##### 3.1.3.3 A long way from home

Student-athlete life (Case 3) demands balancing sport with study requirements, deadlines, and maintaining eligibility to play (19, 86). The decision on which college to attend was a stressful process, with criteria including division, location of the college, scholarship offer, coach compatibility, and expected playing time/style (19). Baller-C explained his choice: “Junior college (JUCO) isn't for everyone … picking the right fit for you is more important than the level or name of the school … it's a good starting point for me”. Attending a lower-division college increases the chances of playing time but often comes with pressure to upgrade to a higher-division school after a 2-year tenure. Baller-C overcame his initial trepidation of the JUCO team's “gangster culture” and was recruited to an NCAA Division-II college after graduation. Pressure for those attending Division-I colleges came from high-profile spectator games, NBA prospects and commercial presence (BB-Manager2). Despite completing his tenure, Baller-A said of his team environment, “We had a new team year after year … You couldn't trust the coaches … it was a toxic environment” (94). Yet, it is in the best interests of college coaches to support student-athletes and ensure team succession beyond the freshman year (18). Basketballers also expressed migration challenges in post-college seasonal transitions, highlighting a sense of homelessness; “I kinda anchor myself with my suitcase” (Baller-A). Baller-B spoke of struggling when he moved to a small non-English-speaking town: “It was kind of a shock” without the support he had in college. Baller-A, playing a season in Europe, “I'm just in a bad mood … We've had lots of imports in and out [of the team and the house], but living in the house keeps me sane. Otherwise, it would not be *yeah* [not good]”.

#### 3.1.4 Coping with adversity

As athletes progress in emerging pathways, the “funnel” of success becomes narrower, and demands accelerate with increasing “sacrifice”, commitment, and physical and mental intensity to progress (35, 95). Coping with adversities, setbacks, or acceptance of career endings were diverse yet painful for all.

Participants in all three cases faced complex and compounding events, relational conflicts, adversities and crises. For example, Boxer-A lost his contract through poor fight decisions, Player-C dealt with a major injury, Player-F changed clubs mid-pathway, Baller-C had conflicts with his coach, and Player-A's partner became pregnant. BB-Analyst noted a significant dropout rate of NZ Collegiate Ballers after the first year, e.g., Baller-A was the only one of eight who finished in his peer group. Homesickness as a common reason for dropping out and feeling isolated. Many others drop out due to frustration with not getting playing positions and time “minutes” or academic struggles (BB-Analyst). “If you don't perform, you're not getting a contract” (RL-Manager2), most commonly, athletes battled with selection events (4, 79), in understanding “why they're on the bench” (BB-Coach1), or unable “to buy” that fight opportunity (Boxing-Manager). For example, non-selection (Baller-J), de-selection (Player-F), moving in and out of starting positions (Player-B and Player-E), and “sitting on the bench (Player-A, Baller-A, Baller-C and Baller-F)”, “just waiting” (Boxer-A).

Some athletes need more structure and support (e.g., Player-E, Baller-C) than others (Baller-A, Boxer-A). Some suffered emotional crises (e.g., Baller-B, Player-F) (93). Athletes who reframed setbacks positively, like Baller-A, who despite his toxic college environment, focused on having “fun with it … cos on game day. I'm living my dream”. Athletes like Player-C hid and/or avoided hard conversations and many early exits “disappeared into regular lives in Australia” (RP). Avoidance coping was common with Pasifika athletes, as failings represented shame “feeling they had disappointed you” and/or “let their family down” [RP, Wellbeing-Manager (85)]. The above examples demonstrate variations of problem, emotional, and help-seeking strategies (11).

##### 3.1.4.1 Coping with endings

When adversities suggest endings, particularly premature or unpredictable ones, anguish is inevitable (43). Significant team-sport events, like injuries, (cases 2 and 3) can result in career endings. However, a prize-fighter may continue to make money as a journeyman until he or his body decides otherwise. In both instances, the individual grieves the loss of who they believed they were destined to be (53). Player-D and Player-G described feeling disillusioned and lost when the club released them at the end of their U20s pathway. Player-D decided to finish his university studies and enjoy playing domestically. After a period “off the rails”, Player-G pursued an opportunity in Australia. However, given substantial mismanaged knee injuries, he was forced to quit. Some years later, he was still emotional at what could have been. After finishing his degree, Player-D was determined to have “no regrets”. “Giving it” “another crack”, he fought his way back into a professional NRL reserve side. For those away from home, the ability to “stick it out” was grounded in their meaning for staying (Player-A, Player-B). Sometimes, endings were observed as self-sabotaging actions or behaviours in younger athletes Being dropped meant they could blame someone else rather than admitting they just wanted “to be a kid sometimes” (Player-E). After suffering two losses, Boxer-A's manager and promoter terminated his contract. Despite being poorly handled and left stranded with visa issues, Boxer-A positively reflected, “It got me to America right” (90, 96) still refusing to accept his career as over, believing anything he “wants will come to him at the right time”—and what he wants more than anything is to be a world champion.

##### 3.1.4.2 Resources for coping

Athlete readiness for events and relocation was variable. Coping depended on circumstances and resources specific to the individual, such as whether parents were accompanying them, destinations and distances, family dependency, domestic capabilities, prior visits, prior connection with others in the team (including the coach), and physical, logistical, and psycho-social preparations. Pre-transition research, transition plans, and talking to other experienced players, parents, experts and practitioners provided an understanding of the conditions, expectations and pressures athletes would experience (BB-Manager1, RP, RL-Manager1). In Case 3, strong mentoring support meant student-athletes (Baller-C, Baller-D, Baller-E, and BB-Manager2's son) were better prepared and counselled on college decisions, logistics and expectations. Club-managed homes or college residential accommodations indicated a helpful buffer for athletes in their initial step away from home. The initial accommodation allowed time to gain independence, with social support (peers, house parents and personnel) in proximity. Perhaps a closer social support presence also enables early identification and action of potential issues. The importance of connections to local people was mentioned by several participants, as providing them with a sense of belonging, feeling part of the community, as well as a knowledgeable network of people to draw on: “One of my best mates was local” (Baller-A) and “fortunate a local family reached out” (BB-Manager2).

As such, these findings demonstrated that knowledge, experience, and preparation strategically facilitate coping with adversities.

### 3.2 “No-one can get in there and fight for you”

Existentially, athletes are always free to *choose* the attitude and exertion they apply to the task (49). They must choose because “No one can get in there and fight for you” (Matchmaker). They decide whether to train and compete on the field or court, get in the ring and fight, advance or withdraw, regardless of age. For example, Player-E was not as “hungry as the other boys” and was deselected after prioritising work over training. Player-A let his actions do the talking, ultimately signing a senior NRL contract at 21-years. This theme responds to question 2, examining the characteristics of *being-an-athlete*, the need for social support and *the inner circle* of people they can trust and who can rely on them.

#### 3.2.1 Being-an-athlete

This theme underlines the investment needed to be a professional athlete goes beyond the physical characteristics or the thrill of competition to the long hours of grind and repetition. Athletes who *choose to be* professional draw upon internal physical and relational characteristics to strive and thrive. In these cases, most participants expressed a strong desire to pursue professional sports careers at the onset. “Sacrifice”, “accountability”, and “work ethic” were the most commonly talked-about criteria for success across all three cases. As Player-D highlighted, “90% of what I really do with my time is train”. Boxer-A stated you “just have to train hard, eat right” and “not get frustrated” Sacrifice, in this instance, refers to committing to forgo other activities and past-times and do what is required to excel in their sport. For emerging athletes, this includes “sacrificing” social pursuits other teenagers engage in (86, 97, 98). In basketball, participants described this as accountability, “You can't be blaming the coach … the team-mate… the roster” (Coach-1) and for the boxing athletes, “it was a sin to go into a fight unprepared” (Boxer-A). These are all examples of where desire and existential responsibility must be matched.

##### 3.2.1.1 Physical characteristics

“Being a gladiator” in boxing implies being “iron-willed”, “savage”, and “dangerous” and prepared to fight to the end (99). Although analogies of entering the battlefield are more overt in boxing, other athletes adopt similar descriptions when entering the field of play. Findings across the three sports referred to athletes' physical presence, being a “warrior”, having “strength and speed”, “being explosive”, having physical size, “being big”, “being tall”, or “being brutal”. Weight and body mass were constantly referred to, particularly in weight-restricted sports like boxing. Physical factors involve the ability to withstand the demands of dieting, training and competing and dealing with the inevitable niggles and injuries. In rugby league, power and strength in contact, playing through injuries, and pre-game rituals represent “going to war” alongside their brothers and include cultural influences from Māori and Pasifika tribal traditions (100). However, they did not always know how to translate their physical attributes into performance. Baller-G needed coaching on “how to be tall” and more aggressive. Furthermore, athletes described the physical attributes outside of sport as a curse—“[I'm] 6′8 … [strangers] ask if I play basketball, so if it was ‘no’ … it *would* affect me” (Baller-C).

##### 3.2.1.2 Performance characteristics

Performance characteristics refer to the athlete's capacity and attributes to develop, train and compete at high-performance levels. To “make it” in professional sports, the athlete must be the best or at least one of the best (49). Findings show successful transitions rely on an athlete's ability to overcome doubts, maintain confidence and work ethic, cope with setbacks, rise to challenges and possess a hunger and commitment to achieve. Successful performances and selections increase confidence. Confidence is established through repetition and experience in training and competition events (101). Ensuring athletic confidence was described as pivotal in pre-fight training camps (Case 1) through planned programming—“Every time you hit a step, you knew, you’re closer to your goal” (Boxer-B)—and knowing they had done the work, for Boxer-C by “ticking off the sparring rounds” (98). As Boxer-C summarised: “It is *not* how big your muscles are, or how fit you are, it's all about how confident you are when you walk in the ring.”

*Confidence* contributes to transition coping in the same way it contributes to performance. Having done the work and prepared adequately for phases and events, athletes were more confident in their skills, character, and ability to adapt and to take the step-up or in moving into new environments: “You are there [selected] for a reason … so you just have to have confidence” (Player-E). Conversely, an athlete's confidence was knocked when they lacked comprehension of the environment or situation they entered: “Player-F thought he was coming into a very different situation” (BB-Coach2), “It was a slave contract” (Boxer-A), “He's upset he was moved out of position” (RL-Manager1), “I wasn't ready to be more independent” (Player-E). Interpreting and resourcing “weekly selection events” took its toll on the athletes' confidence, underscored by Baller-C describing coming off the bench in the closing minutes: “It's a bit of a façade” (Baller-C). Nonetheless, coaches measure players' ability and attitude during these events: “Stay confident and alert with what's going on … then you're ready when the opportunity comes” (BB-Coach1).

*Competitiveness* (including hunger, assertiveness or aggressiveness) was an emotion that athletes were asked (by coaches or the public) to display in some circumstances and put aside in others (102). While there is an element of putting on a mask and playing a role in all three codes, these dichotomies were most evident in boxing. The significance of role-playing in pre-fight marketing and competition is critical to the successful elevation of an emerging boxer. This role-playing, further complicated by the need to be “savage” and entertaining on demand, means “you tell them to hide [their emotions] and at the same time show them” (Boxing-Manager). To sell tickets, the athlete must compete fiercely, “put on a show”, and fight courageously in the spectators' eyes. As Matchmaker stated, “It's all entertainment, but it's dangerous entertainment”. A non-entertaining boxer will not secure a promoter. As Hutchens affirmed, what the public reveres in the ring under institutionalised regulations is abhorred outside it (102). The nature of the boxer is to fight: “It's my excuse to be an absolute dick because I get to beat him up” (Boxer-B). Yet, outside the ring, Boxer-B is an intellectual, reserved and quirky individual (RP). Indeed, boxers see themselves as entertainers, but this is juxta positioned with strength and power.

#### 3.2.2 The inner circle

When people are under pressure, they recruit confidence from trusted sources (103). Participants referred to being in it together “side-by-side”, “being comfortable”, “really loyal … real strong culture”, “being mates”, “if you fail, you fail together”, “someone I trust”, “truthful”, “caring” and “transparent”. This sub-theme represents the importance of relationships in sporting environments, including *organisational relationships*, *team fraternity* and *practitioner relationships,* followed by *authenticity and connection*.

##### 3.2.2.1 Organisational relationships

It would be reasonable to expect organisational personnel to act with integrity and be truthful, and transparent in their approach. “The player just wants to know, what can I do better” (BB-Manager2), “yet there were times I tried to sugarcoat [instead of being] blunt and telling them” (BB-Coach1). Participants spoke of agents who were “dodgy” (Player-D, Wellbeing-Manager) and coaches as: “a guy to definitely favour” (Baller-C), “very hard on you, [ …] they yell a lot” (Player-E), “he's quite an intimidating figure” (Player-D), “They will drop you just like that … here is all about winning” (Player-E).

However, findings showed that transparency and truth-telling were often conflicted, hedged and manipulated to accommodate instituted organisational values, behaviours and actions. Contrary to emerging athletic and personal development priorities, organisational representatives are under pressure from stakeholders to prioritise contracts and mandates to win, attract sponsors and entertain (5). Staff can be conflicted when development sits alongside performance and selection decisions; similarly, players feel vulnerable opening-up about issues (e.g., hiding injuries). Participants explained when money is involved in sports, the dynamic shifts and self-interest increases. For example, we saw BB-Coach1, recruiting emerging ballers to save money and stockpiling players waiting for senior athletes to arrive.

The coach-athlete relationship in boxing was witnessed as more entrenched, “being all in” and juggling between “two relationships”, friendship and business. This relationship is critical as the coach and/or manager must protect the boxers from themselves, make the right fight decisions, and be in the ring to ensure their physical safety (104). This intense bond was described as being “on the battlefield together”, “almost like a marriage”, yet at times RP observed sometimes it bordered on “co-dependency” or “subservience”. Boxer-A stated, “I never say no to these people at any time”. Boxer-B's career confirmed Wacquant's findings (99), operating inside a gym strongly directed by the nature and style of the head coach: “I think you need to keep everything in-house” (Boxing-Coach). Conversely, Boxer-A's story told of an inner circle seemingly disconnected in terms of protection and responsibilities, while Boxer-C referred to needing a small circle: “I didn't have a lot of people around me—I didn't like a lot” just a tight trustworthy team who could be independent and strategic in fight decisions and “to pull me back when I got ahead of myself”. As stakes rose, decisions about who was in the inner circle became muddied with power and finances; with previously trusted advisors told to “stay in their lane” (RP, Matchmaker). Relational authenticity was exposed when the coaches became more controlling: “When multiple people get involved … and once emotions get involved … that's when it becomes complex” (Boxing-Manager).

##### 3.2.2.2 Team fraternity

While coaches valued a strong team ethic, athletes were sometimes conflicted between teamwork, “brotherhood”, and competitiveness (49). Athletes needed to operate or behave as one fraternity (49), demonstrate commitment, enact selflessness for the team's betterment, or to secure wins. However, when observed closely, teamwork or cohesion (including among staff) was more tenuous when positions were at stake, or sides were losing. The 18th man in rugby league is eager to take the field but only plays if a team-mate gets concussed, his best mate plays in the same position, or (BB-Coach-1) vs. “feeling like you have been going harder than the ones getting more court time in front of you.” (Baller-to-Executive). The individual must mask their true nature, frustrations and feelings to fill their designated public role (athlete, coach, etc.) “sitting on the bench shows you are committed to the group” (5, 29). Here, fraternity contradicts the competitive nature of the individual, who wants to prove themselves better than others for coveted positions, starting sides, winning points and lucrative contracts. In boxing, fraternity differs; when they step into the ring alone, the boxer accomplish wins alone. They don't have “to share the accolades with team-mates who may not equally deserve them” [(99), p. 515]. Still, they rely heavily on their corner to “jump in” (Boxing-Coach) and protect them (104).

##### 3.2.2.3 Practitioner relationships

Critical factors in facilitative practitioner supply included the importance of lingering with intent—being present (available, familiar and reliable), having rapport, trust, affection for each other, with athletes adding the importance of having “someone who believes in me” (Player-E), “having their back” in good times and bad and investing time and effort towards their successes. Confidence is crucial in the heat of competition and is often grounded in ego. In boxing, ego is a requirement to fight to survive and win (59). As RP experienced, emotional and often aggressive outbursts occurred pre-fight when the boxers and coaches were most vulnerable. The practitioner's role demands they set aside their feelings to protect others' egos. Generally, athletes want someone to vent or talk to (105): “A lot of the times, players don't want to take it any further; they don't want the coach to know about it. They just want someone to know how they are feeling” (Baller-to-Executive). For Player-A, it was the administration lady who would give him a hug and encouragement, whom he described as the “heart of the club”. RP concluding, “Small things do make a difference”.

##### 3.2.2.4 Authenticity and connection

To succeed, individuals on both sides of relational dyads must fit into the organisational systems and culture and enact stipulated institutional demands (49), diminishing them to a state of *situated freedom* (80) Individuals may think or behave differently to become accepted by others or to achieve a particular result, acting inauthentically or in bad faith. These findings showed failure to feel *connected* can be a source of crisis for some athletes (and other stakeholders). Even inside team environments, it can still be a lonely and isolating proposition: “By the end of the season, [Baller-C] did not care, and no one cared to try and talk or help him through” (BB-Coach3). Athletes' needs (perceived or actual) for social support was consistently demonstrated across all three cases. Novelly, the importance of modern-day electronic messaging (texting, social media channels) was now an essential mechanism for prompting and checking-in, reminding adolescent athletes you are there (RP). *Presence* availability and reliability, physical or electronic, are fundamental requirements of facilitative relationships in these cohorts, as well as mutual investment and respect for each other's contribution (59). Boxing-Manager explains the need for mutual investment: “I am not going to waste my time … your shelf life is limited, and that goes for the boxer and the coach”. RP (like Matchmaker) “felt used and genuinely grieved not being part of the inner circle”. Later, accepting “the ending was an organic part of the process” (RP, Case 1). “Small things matter” for the supplier too: “getting a reply to my text even if it was just an emoji” (RP), “being thanked” when I go beyond (BB-Manager1), “he just didn't hear what I had to say” (Matchmaker).

## 4 Discussion

Section 4 focuses on two areas of discussion: firstly, coping with career transitions, and then expands further into existential perspectives—meaning (including connection), choice, and responsibility.

### 4.1 Coping with career transitions

Figure 2 presents the final conceptual model formulated from multi-case data analysis. This model is designed to provide a comprehensive view of the processes and interactions for the EACT. Figure 2 captures career transitions as a coping process (11), commencing with events influenced by transitional pre-conditions, responses, and outcomes impacted by appraisal/adaptation.

EACT comprises different phases in different organisational environments but typically includes identification, development (TDE/Collegiate), and Junior-to-Senior (within senior). Across these phases, athletes experience multiple events: selections, terminations, injuries, migrations, relational conflicts, and contracting. For some, the Junior-to-Senior step may be notional; for others, such as Player-A, it can be a lengthy apprenticeship, which may or may not result in desired outcomes of professional senior contracts. Events experienced by emerging athletes are categorised as *transition*, *performance* (athletic and personal development) and *relational demands*. Events rarely occur singularly (*complexity*) but are more commonly embedded (e.g., de-selection and coach conflict and/or injury) and not all events are bad (e.g., selection, contracting). Events are tagged by form (negative <positive), intensity (including controllability) and complexity (singular <multiple) (106).

Outcomes comprise physical, mental and emotional fitness, freedom and connection, and performances in sport and life. The findings recorded physical, mental or emotional fitness fluctuations over time, with participants moving across the continuum of outcomes from crisis (e.g., Player-F, Boxer-C), striving (e.g., Boxer-A, Baller-C) to thriving (e.g., Player-A) *Freedom* allows individuals to operate in “good faith” (transparently and true to self). *Connection* recognises that “both the provider and the recipient bear responsibility for cultivating effective support relationships” [(59), p. 130] through authenticity and presence.

An athlete's ability and strategies for coping were predicated on the time, context, and attributed meaning given to their sporting career (e.g., Player-D entry into university). The response to the situation/event reflected the intensity factor (e.g., Player-C vs. Player-G injury) and the athlete's interpretation [Baller-A “just blocked (toxic coaches) out”]. These appraisals reflected how athletes chose to adapt, whether they saw it as a challenge or an uncontrollable threat, determined by continued “work-ethic”, “patience and persistence”. Adaptions support actions in crisis transitions (6); decisions to act (e.g., Boxer-C still fighting for the chance to be world champion), accept (e.g., Baller-C sitting on the bench), or reject (e.g., Baller-J withdrawing). We also found that shame, particularly with Pasifika athletes, was demonstrated by the avoidance of conversations (including lying) or withdrawal physically and/or from significant others (107, 108). Consequently, the proposed model recognised self-efficacy and foundational resources (relational and physical) as necessary conditions for coping.

*Self-efficacy,* defined as *situational confidence* in one's abilities and the certainty one can and will make the sacrifices (*commitment*) necessary to do whatever it takes to overcome adversity (*resilience*) and succeed (109), provides a robust facilitator to deal with the pressures and setbacks in sport and life. Cross-case findings revealed: “Confidence” enhanced through preparation and readiness (e.g., Boxer-C “ticking off the sparring rounds”), belief (in themselves and having “someone who believes in them” and resiliency (being “tough” and “committed”) and robust enough to “grind through” setbacks. Self-efficacy warrants further quantifiable research; the construct arguably supporting coping capabilities (110) *and* performance (111). Lochbaum et al. found evidence that pre-event self-efficacy has a meaningful impact on sports performance, with higher correlations found in elite sports.

Regardless of an athlete's internal resources, athletes across these cases drew on their “inner circle” for instrumental and/or emotional support. Perceived or actual uptake of social support was more successfully facilitated through the supplier's presence (proximity and reliability) and authenticity; “small things matter” (RP). Findings also supported the need for mutually beneficial relationships (59), “coach was willing to make it happen because he was trying to build something for himself too” (Boxer-B). Authentic relationships exist when each person sees and respects humanity in the other (51). Conversely, authentic relationships were undermined when transparency, truth, trust or genuine care were questioned.

Finally, while previous researchers have defined the anguish of career endings as “athlete identity foreclosure” (48), this study posits crisis outcomes are better described in existential terms. These findings argue career crises/endings are akin to the death of their dream (49): the individual grieves the loss of who they believed they were destined to be (49). To these young athletes, the world only has meaning as far as it opens to a potential future, in which their hopes are still committed to “being-a-professional-athlete”, a dream many had held since they were small children. The following sections expand on existential perceptions as preconditions for EACT.

### 4.2 Existential perspectives—meaning

Unpicking the meaning athletes assign to pursuing a career in sport requires delving into culturally accepted and automated responses. “*Meaning* is shared mental representations of possible relationships among things, events and relationships … Meaning connects things” [(112), p. 466]. Furthermore, meanings are rarely singular, often embedded and difficult to separate as unique constructs. Adolescent athletes typically retort, “I want to be in the NRL,” or say they are doing it “for my family”. Is it a financial thing, is it to make their family proud, are they trying to follow in a family member's footsteps or is it a completely different “why?”. The desire to help the family, make them proud, and contribute to the collective was especially true for Pasifika families. However, with family honour also came expectations and pressure to uphold family security and pride (3, 107, 113). Pasifika athletes, one to two generations removed from the Islands, saw themselves as “Kiwi kids” (114), with identity and meaning spliced between traditional family and modern urban youth cultures.

We found the meanings expressed in these cases fell within four constructs: *a desire to better oneself and their family* (security, money*), the desire to prove oneself* (status, prowess, and success), and *the desire to do something of significance* (to mean something in the world) and the *desire to feel connected (to others)*. The first three align with Wacquant's research (99).

#### 4.2.1 To better oneself

The desire to better oneself and one's family was more apparent in the athletes from less secure financial backgrounds: “To buy my parents a house”. Even contracts that included only small amounts of “money to feed” could subsidise their households. The potential for the amounts paid to NRL players were significant. Basketball participants described the meaning for financial security in terms of its “vulnerability” of income stream. When player payments were late, team resentment was evident (RP), so making a living for these professionals was a minimum requirement. Conversely, where one might expect scholarships to represent a priority in terms of free education for collegiate athletes, Case 3 participants inferred more meaning to the opportunity to experience going to a US college and playing. Boxing stakeholders emphasised the difficulties in financing emerging boxer campaigns. The notion of security was also implied by Boxer-B migrating from Africa, stating, “It got me to America”, but Boxer-B's desire to be a world champion and prove himself successful exhibited as great a meaning as security and money.

#### 4.2.2 To prove themselves

Predominately, athletes found meaning in their love of the game, the thrill of combat, and their “hunger” to represent, compete and win (49); having permission to be aggressive proved themselves to be “warriors” (102). Boxers yearning to be a world champion was closely related to expressions of being known as someone who “knew how to work hard more than anyone” (Boxer-C). In Cases 2 and 3, participants spoke of wanting to impress and look good in front of their peers, a norm for their adolescent development stage (57). Interestingly, the broader prestige of fame seemed a more comfortable proposition with the boxers, perhaps linked to the need to be an entertainer. Individual accolades or fame were expressed as less of a priority for some, albeit a nice by-product: “Little kids saying hi to you … is really cool” (Player-A), “I will take pictures with some kids after the game, but I am not into it [when it impacts personal space]” (Baller-A). Both these players were humble Pasifika athletes, which may have a bearing on their responses. It is interesting how both appear more comfortable with children admiring them, intimating they were less comfortable with adult fans. There was a sense that fandom was something that contradicted their humility rather than holding no meaning.

#### 4.2.3 To do something of significance

This meaning showed in the reverence players, ballers, and boxers held for “being-an-athlete”. Being a professional athlete was seen as a badge of honour enduring beyond emerging into retirement, with specific organisational representation considered of even greater significance than other representative honours. The pride was evident when players “put on the jersey”, representing successful team selection of worth. The significance, amongst other personal identifiers, externalises the meaning they give to their sacrifices and effort: having converted “having-a-talent” to being one (49). For instance, even in Player-G's failure to achieve and his distress at his lost potential, he reflected, “I was playing injured, but I was playing at a high level … alongside NRL players, and I was playing full games”. For Boxer-C, “I got to spar Mike Tyson. This was a massive opportunity”—Was it scary? RP asked, “No! (pause) Oh yeah, no, no. Oh yeah, I can't really say it wasn't scary”.

#### 4.2.4 Connection

In Pasifika culture, *feeling connected* is known as Vā. It is the physical, emotional, and spiritual connection to people, places, and things (107). Vā offers a unique cultural context highlighted by the Pasifika athletes in these findings. However, the need for connection was not isolated to the Pasifika athletes. Athletes in all three cases spoke of a sense of meaning derived from being with their mates, of being part of something in “going to war together” (boxing), a “brotherhood” (rugby league) and “the strength of the pack” (basketball). The desire for connection and meaning is relevant not only for athletes but for all stakeholders. Participants accenting a sporting endeavour has no meaning if done alone.

### 4.3 Existential perspectives—choice and responsibility

Exercising choice and responsibility becomes a constant process of effort, performance and coping from identification to the end of athletes' careers. Failure to fulfil the institutional demands of their chosen sport results in non-selection and/or termination of contracts. Participants spoke of either sacrifice or accountability: “If you really want to achieve … you got to have sacrifices … at times, it is tough” (Player-A), and “You felt a responsibility to do the right thing and have people hold you accountable” (Baller-B). Here, sacrifice and accountability are congruent with choice and responsibility; drawing out this connection offers freedom rather than restriction. Sacrifice implies hardship and denial; choice suggests personal control and a willingness to accept responsibility for these choices (43, 49).

To “make it” in professional sports, the athlete must be one of the best. To be the best, the athlete must be comfortable standing out above others and must fully commit to developing the requisite physical and psycho-social capabilities to get there. Yet even after making this choice and forgoing all other opportunities, the athlete may still fail (49). In doing so, they must also accept responsibility for outcomes: like Boxer-A, having signed the contract, was left to “[control] every emotion because I am responsible” and Player-D “I went in with my eyes open … no regrets”.

Increasing adolescent athletes' existential awareness of their *freedom* to choose where and how they apply themselves will enable them to move beyond coping to thriving and take responsibility for their successes or failures in life and sports. To achieve this awareness, personnel working with the athletes should use real-time situations as opportunities to point out personal development (115), being mindful of the language used and being direct and truthful about potential and the normality of fluctuating levels of fitness (physical, mental, and emotional capabilities over time) (52). Best practice TDEs go beyond an athletic approach to incorporate ongoing personal development (5).

In summary, the individual's ability to thrive in EACT was shown to be impacted by pre-conditions of time and context, individual existential perceptions (meaning, choice and responsibility) and self-efficacy (enhanced by prior experience, exposure and readiness). When the athlete underpins self-efficacy with a solid meaning for career pursuit, their ability to cope in high-pressure environments is more likely to contribute to successful or at least more positive outcomes (e.g., Player-A, Player-D, Baller-C).

## 5 Research quality and limitations

A common criticism of qualitative case-study research is its perceived lack of transferability. This article's grounding in NZ cases may limit cross-case transfer; however, NZ is not unique in including collectivist cultures. Pragmatists argue that common patterns or insights can emerge when the case is tightly bound, the proposition is well-defined, and data sources are stratified (69). Qualitative multi-case studies provided a valuable methodological foundation for interpreting sports environments. It allowed the researcher (within defined boundaries) to focus on social interactions and explore how different participants interpret meaning, events and relationships in the existing system. The research paradigm recognises the existence of multiple individual realities across and within participants, stakeholders, and cases (67). This methodology provided a solid foundation for formulating a “single reality” using subjective and objective lenses (65). Combining multi-case and stratified data sources provided a robust and pragmatic methodology for exploring the commonalities and differences within and between cases. As such, it offered meaningful patterns in this complex social setting (67, 69).

However, the research was not without its limitations. The pragmatic scope of the researcher-practitioner's practice directed the selection of cases and participants to those she was associated with. That is, male athletes and associated professional sporting networks. While being a researcher-practitioner offered greater intimacy and knowledge of the participants, data, and research subject, there was also some potential risk of over-familiarity. This insider approach and the purposive selection of cases and participants may have resulted in unintentional bias. To minimise bias, a robust process of critical, independent reviews and coding validation was implemented. This design offered further rigour through constant questioning, revisiting, and challenging oversimplification (66).

While this article extends geographical, transitional events and cultural dimensions along with sports codes and organisational environments, the scope for further research into athlete career discourse remains broad. The article endorses Stambulova et al.'s (15) call to extend the athletic career discourse to examine further diversity (age, gender, ethnicities, roles) and scope (refer Table 1) and argues that using a pragmatic case study approach over a longitudinal timeframe provides richer data in a phenomenon that spans months if not years.

**Table 1:** Future research opportunities in EACT.

Athlete career transition	Existential being	Social support relationships
• Advance longitudinal case-based research in emerging athlete transitions across professional sports and countries. • Incorporate case research to evaluate the implementation of programme changes and interventions. • Qualitative analysis of transition outcomes (commencement to exit). • Further clarity to pathways within different sports athlete career discourses. • Advance research on “parents are transitioning too”	• Continue to evolve an existential perspective in the investigation of athlete lives in and beyond sport. Examine well-being as a construct in relation to existential being and culture, while retaining the concept of a continuum from crisis to thriving	• Continued focus on social support research as a source of strength in the facilitation of athlete and personal development, alongside advocacy in difficult times and crises (13) • Investigate alternative and modern modes of social support, e.g., texting

## 6 Conclusion and recommendations

Sartre considered efforts to reduce the complex personality of an adolescent down to a few basic desires as naïve, so to gain some understanding of the person, we must go beyond categorisations to reveal their “transitions, the becoming, the transformations”, most of which are carefully masked from us [(48), p. 726]. Over the length of this study, individuals’ identities emerged as multi-faceted, dynamic, and socially derived beings (45). These findings concur with Sartre: Greater importance should be given to athlete awareness towards choice, meaning, and responsibility rather than dual career development and identity protection. There is greater benefit to understanding the developmental transformations of emerging athletes and how these culminate in thriving levels of fitness, freedom, connection, and performance. Most emerging athletes will terminate their sports careers in their late teens or early twenties and still have plenty of time and opportunity to pivot. As such, we argue it becomes less about hedging with an alternative career option during their sports career apprenticeship and more about ensuring scope to develop a broad base of relationships and experiences. The importance here is to provide developmental resources and to open minds to alternative education and career opportunities when they are ready and need them.

We further conclude knowledge is power: support is facilitative. Athletes (and their families, if underage) must take responsibility to arm themselves with the right resources to deal with professional sports demands and expectations. Emerging athletes must know where and how to access the necessary knowledge and advocacy and not rush into entering contracts without sufficient due diligence. Improving the integration of development in pathway programmes will offer better knowledge and support to athletes, parents, and staff. Additionally, athlete-player associations should represent a more substantial presence in emerging athletes’ support and transition, especially early exits.

Critically, practitioners and organisational representatives must remember emerging athletes are not adults. RL-Physiotherapist summarises: “We need to remember that some of these kids are 15, 16, 17 … you can't expect a 17-year-old to do it all on their own”. In high-pressured sporting environments, connected, mutually beneficial, authentic relationships indicate better opportunities for individuals to develop and thrive (59, 116). This research found little evidence of personal development alongside athletic development, particularly in preparation for or following termination. We argue that offering a broader range of practitioner skillsets (including sports psychology) integrated inside “the inner circle” as opposed to a separate silo or provided as the “ambulance at the bottom of the cliff” is recommended. As BB-Coach3 emphasised, an ideal environment would be one where different personnel come together with diverse knowledge and skills, offering different perspectives on athletic and personal development.

In conclusion, a conducive environment can aid the development of coping strategies when faced with the inevitable setbacks athletes experience during their careers. Fostering authentic, collaborative cultures in emerging athlete environments that embrace athletic and personal development objectives will “concurrently ensure positive impacts and minimise predictable negative outcomes [for emerging athletes] without losing focus on a drive for sporting performance” [(117), p. 1].

## Data Availability

The data analyzed in this study is subject to the following licenses/restrictions: data is stored conforming to AUT Ethics Committee Guidelines. Requests to access these datasets should be directed to patricia.lucas@aut.ac.nz (Dr. Patricia Lucas).

## Footnotes

^1^Professional athletes are defined as those who primarily dedicated their time to training and competing in return for financial remuneration or subsidy (scholarships). Professional sports scope was extended to collegiate scholarship programmes, particularly given recent developments around name image rights (2).

^2^Māori are the indigenous people of NZ, and Pasifika (or PI or Polynesian) is an umbrella term used in NZ for descendants of the Pacific Islands (e.g. Samoa, Tonga, Fiji).

^3^Organisations being a collective of individuals combined by artefacts.

^4^Long D. *Moneyball: Battle for Footy Talent Getting Younger.* Stuff (2018). Available online at: https://www.stuff.co.nz/sport/rugby/provincial/103297494/moneyball-battle-for-footy-talent-getting-younger

^5^Contenders are contracted boxers who have a pathway to a lucrative championship fight.

^6^Whitebaiting is fishing for and netting lots of tiny bait fish called whitebait.

^7^Colloquial term used to describe New Zealanders.

^8^The ditch refers to the Tasman Ocean between NZ and Australia.
